# Evaluating consistency of recall of maternal and newborn care complications and intervention coverage using PMA panel data in SNNPR, Ethiopia

**DOI:** 10.1371/journal.pone.0216612

**Published:** 2019-05-09

**Authors:** Linnea A. Zimmerman, Solomon Shiferaw, Assefa Seme, Yuanyuan Yi, John Grove, Claire-Helene Mershon, Saifuddin Ahmed

**Affiliations:** 1 Department of Population Family and Reproductive Health, Johns Hopkins Bloomberg School of Public Health, Baltimore, MD, United States of America; 2 Department of Reproductive Health and Health Service Management, School of Public Health, Addis Ababa University, Addis Ababa, Ethiopia; 3 Department of Reproductive Health and Health Service Management, School of Public Health, Addis Ababa University, Addis Ababa, Ethiopia; 4 Department of International Health, Johns Hopkins Bloomberg School of Public Health, Baltimore, MD, United States of America; 5 World Health Organization, Geneva, Switzerland; 6 Bill and Melinda Gates Foundation, Seattle, WA, United States of America; 7 Department of Population Family and Reproductive Health, Johns Hopkins Bloomberg School of Public Health, Baltimore, MD, United States of America; National Institute of Public Health, MEXICO

## Abstract

**Background:**

There is recognition that effective interventions are available to prevent neonatal and maternal deaths but providing reliable and valid coverage estimates remains a challenge. Household surveys rely on recall of self-reported events that may span up to 5 years, raising concerns of recall bias.

**Objective:**

This study assessed the reliability of maternal recall of pregnancy, delivery, and postpartum events over a six-month period and identified relevant individual characteristics associated with inconsistent reporting.

**Methodology:**

A longitudinal household survey was conducted with 321 pregnant women in 44 enumeration areas in Southern Nationals, Nationalities and People’s Region in Ethiopia. Women who were six or more months pregnant were enrolled and interviewed at seven days, six weeks, and six months post-partum using an identical set of questions regarding maternal and neonatal health and receipt of select neonatal care interventions. We compared responses given at 7 days to those reported at 6 weeks and 6 months and conducted sensitivity, specificity, area under receiving operative curve, and Kappa analyses of selected indicators.

**Results:**

We find that reporting complications is higher at the first interview after birth than at either the six-week or six-month interview. The specificity of the majority of complications is high, however sensitivity is generally much lower. The sensitivity of reporting any complication during pregnancy, delivery, or post-partum ranged from 54.5% to 67.6% at the 6-week interview and from 39.2% to 63.2% at the 6-month interview. Though sensitivity of receipt of neonatal interventions was high, specificity and kappa demonstrate low consistency.

**Conclusion:**

As with childbirth, it may be that during the first seven days women note symptoms with higher scrutiny, but if these do not later develop into serious health issues, they may be forgotten over time. Maternal complications and care are likely to be under-reported by women if interviewed for distant events.

## Introduction

There is recognition that “we know what works” to prevent neonatal and maternal deaths and that proven, effective, and cost-effective interventions are available [1,1–20]. Though health management information systems (HMIS) can be used to track coverage of some interventions, the majority of data on interventions received through community outreach or in the home rely on household surveys such as the DHS and MICS [21]. Due to this reliance on household surveys, a growing body of research in low-and middle-income countries (LMICs) is assessing the validity of self-report of receipt of interventions.

The majority of reproductive, maternal and newborn care health (RMNCH) validation studies assess the consistency of recall between facility based interventions and women’s subsequent report and find that generally the ability of women to report accurately depends on the intervention itself, on the delivery experience, on characteristics of women, and on the time elapsed since receipt of services [21–24]. Recent research also suggests that the ability of women to recall and report pregnancy and delivery events is also influenced by the type of measures; concordance between maternal recall and obstetric records for continuous measures (such as gestational age and birthweight) may be better than dichotomous measures (such as the presence or absence of specific delivery events)[25].

To address sample size challenges, the majority of household surveys rely on women retrospectively recalling birth events over a two to five-year window. Evidence has been mixed on whether the recall period has an effect on the ability of women to report accurately [26,27]. While recent work by Moran and colleagues found that at the population level, reporting of some indicators did not seem to be affected by time since birth, they were not able to assess whether individual responses were consistent over time due to the cross-sectional nature of household surveys [28]. Stanton and colleagues found that recall within a twelve-month period was problematic, while Stewart and colleagues found no relationship between the recall period and the validity of women’s report. None of the studies, however, reviewed consistency of reporting over time and whether consistency of reporting varied by time, indicator, or background characteristics of the respondent.

### Country context

In recent years, Ethiopia has put forth substantial effort to address maternal, neonatal and under-5 mortality, but maternal and newborn health service utilization remains low. In Southern Nations Nationalities and Peoples Region (SNNPR), the site of this research, approximately 30.4% of women who had a birth in the five years before the survey received no antenatal care, 73% delivered in their home and 19% received any postnatal care [29].

Utilizing a longitudinal study design, the Performance Monitoring Accountability 2020 Maternal and Newborn Health (PMA-MNH) study was conducted in Southern Nations Nationalities and Peoples Region (SNNPR) of Ethiopia to monitor the use of proven interventions to reduce maternal and neonatal mortality. A battery of questions were repeated at each interview to assess consistency in maternal recall of events surrounding the pregnancy, delivery, and post-partum period. Using these data, we intend to: assess the reliability of maternal recall of pregnancy, delivery, and postpartum experience of complications through self-report of symptoms; assess the reliability of maternal recall reporting neonatal care received in the immediate postpartum period; assess the reliability of maternal recall reporting neonatal illnesses experienced in the first seven days of life; and identify relevant individual characteristics associated with consistent, and conversely, inconsistent recall of events

## Methods

### Study design

The study design was a longitudinal household survey to collect knowledge, practice, and coverage information of maternal and newborn care interventions. The study utilized the existing survey platform of PMA2020, which has been implemented in Ethiopia since 2013. PMA2020 is a survey platform, operational in 11 countries, that trains and employs local women to serve as enumerators, conducting face-to-face interviews on smartphones to generate rapid turn-around data [30]. In Ethiopia, PMA2020 is a collaboration between Johns Hopkins Bloomberg School of Public Health (JHSPH), Addis Ababa University (AAU), the Federal Ministry of Health (FMoH) and the Ethiopia Public Health Association (EPHA).

The enumeration areas for PMA2020 were selected using a two-stage stratified cluster sampling design and selected with probability proportional to size within urban and rural strata. At the time of the launch of the PMA-MNH survey, PMA2020 had conducted four rounds of data collection in 47 enumeration areas (EAs) in SNNPR. After considering the logistical challenges of conducting longitudinal data collection, three enumeration areas were dropped, resulting in a total of 44 enumeration areas participating in the initial screening of households for PMA-MNH. One enumeration area had no reported women who were 6–9 months pregnant. This was verified by the supervisor in the field and was subsequently dropped from the longitudinal follow-up. The final 43 EAs were included for PMA-MNH.

### Sampling strategy

The study team first conducted a complete census of the 44 EAs and conducted a brief household survey. All household members were enumerated and all women between the ages of 15–49 were screened. Women who were six or more months pregnant, by self-report of duration of pregnancy, were eligible to participate in the longitudinal study. A household and individual questionnaire were completed at the time of enrollment. During the postpartum period, resident enumerators (REs) returned to administer questionnaires in-person at seven days and six weeks postpartum, and either called or returned in person at six months postpartum to administer the final questionnaire. REs obtained the contact information of women during enrollment and maintained frequent communication during the study period, particularly during the anticipated delivery date. A set of questions regarding maternal symptoms experienced during and after pregnancy, neonatal symptoms experienced during the first seven days of life, and receipt of select neonatal care interventions was asked at each interview to evaluate consistency in reporting. Women were not asked to report on specific diagnoses they may have received either for themselves or their infants, only on specific symptoms that they experienced.

### Ethical clearance

Ethical approval for this study was granted by the Johns Hopkins Bloomberg School of Public Health and the Ethiopian Public Health Institute (EPHI) Institutional Review Boards. Verbal consent was obtained from participants. The IRB approves verbal consent procedures (without a need for written consent) for simple surveys without any invasive procedures in an environment where literacy is low. Detailed information about the ethical guidelines required by EPHI can be found in the National Research Ethics Review Guidelines (http://www.ccghr.ca/wp-content/uploads/2013/).

Table 1 shows the number of women and the number of live infants for which information was collected at each interview. Overall, response rates were high; 97.6% of all women originally enrolled completed the final interview.

**Table 1:** Results of household, female and MNH screening 7-day, 6-week and 6-month postpartum interviews (unweighted).

Number of households interviewed and response rates	
**Household interviews**	**Total SNNP**
Households interviewed	10,140
Household response rate	98.7%
**Female screening interviews (women ages 15–49)**	
Number of eligible women interviewed	9,713
Eligible women response rate	98.4%
**MNH Screening (Pregnant women ages 15–49, in the third trimester)**	
Number of eligible women enrolled in MNH	329
% consented	100.0%
**MNH Interview 1 (7 day postpartum)**	
Number of enrolled women interviewed	324
MNH1 response rate	98.5%
Number of live births	321
Number of neonates alive at 7-day interview	316
**MNH Interview 2 (6 week postpartum)**	
Number of enrolled women interviewed	322
MNH2 response rate	97.9%
Number of neonates alive at 6-week	311
**MNH Interview 3 (6 months postpartum)**	
Number of enrolled women interviewed	321
MNH3 Response rate	97.6%
Number of infants alive at 6-month	309

### Analysis

We evaluated the extent of recall bias in the reporting of maternal and neonatal complications and care received by testing the sensitivity, specificity and area under receiving operative curve (ROC) of selected indicators. We treated the seven-day interview as the standard and compared the consistency in responses at the six-week and six-month interviews. Comparisons of responses to identical questions at each survey round were performed for women who participated in both the seven-day interview and the respective follow-up interview; thus, the six-week interview has 322 responses and the six-month has 321. Sensitivity shows the reported positive responses at the seventh day postpartum interview that were correctly recalled as positive at 2^nd^ and 3^rd^ follow-up visits on 6-week and 6-month interviews while specificity shows the negative responses at first interview correctly reported as negative at follow-up interviews. A convenient way to summarize these test measures is expressed in the area under the ROC. An area of 1.0 represents a perfect recall (test of matching responses) and an ROC area of 0.5 represents an unreliable response. In addition, we used two other measures of assessing agreement: Cohen’s kappa coefficient and agreement in diagonal cells. Kappa is considered more robust than the agreement measurement.

Finally, we assessed whether specific respondent characteristics (age, residence (urban/rural), education, parity, facility delivery, receipt of caesarean section, and a visit from an HEW in the first seven days postpartum) were associated with inconsistent reporting of any complications or illnesses and for three high impact interventions (whether the baby was wrapped, whether breastfeeding was initiated within one hour of delivery, and whether the baby was placed naked on the mother’s chest immediately after delivery). A binary variable for each event (either reported complication or receipt of intervention) was created to indicate whether the respondent reported consistently across all three interviews or was inconsistent in one interview and a multivariable logistic regression analysis run to assess if any sociodemographic variables were associated with inconsistent reporting. Variables were categorized as follows; age into three age groups (15–24, 25–34, 35–49), education into three groups (no school, any primary, and any secondary or above), parity into two groups (first birth versus higher order), and home delivery versus any facility delivery.

Survey weights were not applied in this analysis except to demonstrate representativeness of the sample characteristics when adjusted for the complex survey design. All analyses were conducted using Stata 15.1.

## Results

Background characteristics of women collected during the initial screening and first follow-up interview at 7-day postparum are summarized in Table 2. The majority of respondents were married (97.1%), between the ages of 25 and 34 (51.8%) and had given birth to at least four children (54.0%).

**Table 2:** Sample characteristics of women enrolled in PMA-MNH study (weighted).

	n	%
**Household Characteristics**	**329**	
Wealth group		
Poor	123	37.4
Middle	110	33.5
High	96	29.1
Drinking water source		
All improved sources	232	70.5
At least one unimproved source	97	29.5
HH uses improved sanitation source	30	9.0
HH has insecticide treated bednet	149	45.4
**Respondent Characteristics**		
**Age group**		
15–24	108	32.8
25–34	170	51.8
35–49	51	15.4
**Proportion married**	319	97.1
**Urban**	37	11.3
**Parity**^**†**^		
1	67	20.7
2–3	82	25.4
4 or more	175	54.0
**Educational Attainment**^**†**^		
Never attended	146	44.9
Primary	144	44.3
Secondary/Technical/Higher	35	10.8

† Data collected at 7-day postpartum interview, n = 324

### Maternal complications

Table 3 below shows the numbers of women reporting any maternal complications during pregnancy, delivery, and immediately post-partum. Only those women who reported a complication were asked if they sought treatment, thus among women who reported a complication during only one interview, their report of care-seeking is missing in the comparison interview. This restriction significantly reduced the number of women who contributed information for whether treatment was received, particularly for post-partum complications.

**Table 3:** Frequency of reporting of any maternal complications during frequency, delivery, and post-partum between 7-day and 6-week and 6-month interview.

		6-week		6-month	
	**7-day**	Yes	No	Yes	No
Pregnancy complications	Yes	115	55	106	64
	No	17	135	16	135
Delivery complications	Yes	69	56	61	64
	No	15	182	19	178
Post-partum complications	Yes	53	46	37	61
	No	27	196	28	195

#### Complications during pregnancy

As shown in Table 4 and Table 5, more women reported experiencing complications during pregnancy at the seven-day interview than at the six-week interview and six-month interview for all complications. Specific complications are arranged in order of lowest to highest sensitivity. While 52.8% of women reported at least one pregnancy complication at the seven-day interview, only 41.0% reported any during the six-week follow-up interview and 38% reported any during the six-month interview.

**Table 4:** Agreement diagnostics for select maternal pregnancy, delivery, and post-partum complications reported at 7-day and 6-week follow-up visits.

Variable	N	Reported at 7 days (%)	Reported at 6 weeks (%)	Sensitivity (95 CI)	Specificity (95 CI)	ROC (95 CI)	Agree-ment (%)	Kappa (95 CI)
**Pregnancy complications**								
Vaginal bleeding	322	4.3	1.6	28.6 (8.4–58.1)	99.7 (98.2–100.0)	0.63 (0.52–0.75)	96.3	0.39 (0.29–0.48)
Fever	322	13.7	11.2	45.5 (30.4–61.2)	94.2 (90.9–96.7)	0.69 (0.62–0.77)	87.3	0.42 (0.32–0.53)
Convulsion	322	7.5	7.5	45.8 (25.6–67.2)	95.6 (92.8–97.7)	0.71 (0.61–0.81)	92.0	0.42 (0.31–0.52)
Migraine	322	23.3	14.9	46.7 (35.1–58.6)	94.8 (91.3–97.2)	0.71 (0.65–0.77)	83.7	0.47 (0.37–0.58)
Abdominal pain	321	21.8	14.9	50.0 (37.8–62.2)	94.8 (91.4–97.2)	0.73 (0.67–0.79)	85.2	0.51 (0.41–0.62)
High blood pressure	309	4.8	2.9	60.0 (32.3–83.7)	100.0 (98.8–100.0)	0.80 (0.67–0.93)	98.1	0.74 (0.63–0.85)
Edema	322	18.6	15.8	68.3 (55.0–79.7)	96.2 (93.1–98.2)	0.83 (0.77–0.89)	91.1	0.68 (0.59–0.81)
Any complication	322	52.8	41.0	67.6 (60.1–74.6)	88.8 (82.7–93.3)	0.79 (0.74–0.83)	77.9	0.56 (0.46–0.67)
Received treatment	115	65.9	58.3	75.3 (64.2–84.4)	71.1 (54.1–84.6)	0.73 (0.64–0.82)	73.9	0.44 (0.26–0.62)
**Delivery complications**								
Leaking membrane <9m	322	2.1	0.6	14.3 (0.4–57.9)	99.7 (98.3–100.0)	0.57 (0.43–0.71)	97.9	0.21 (0.12–0.3)
Leaking membrane 24hr	322	7.0	3.7	21.7 (7.5–43.7)	97.7 (95.3–99.1)	0.60 (0.51–0.68)	92.3	0.25 (0.15–0.35)
Malposition	321	3.4	2.5	50.0 (18.1–81.3)	99.4 (97.7–99.9)	0.77 (0.62–0.92)	97.8	0.62 (0.51–0.73)
Excessive bleeding	320	19.0	13.5	53.2 (40.1–66.9)	95.8 (92.6–97.9)	0.75 (0.68–0.81)	87.7	0.55 (0.44–0.66)
Prolonged labor	322	20.1	13.5	55.4 (42.5–67.7)	96.9 (94.1–98.7)	0.76 (0.70–0.82)	88.7	0.60 (0.49–0.70)
Any complication	322	38.8	26.2	55.6 (46.4–64.4)	92.4 (87.8–95.7)	0.74 (0.69–0.79)	78.1	0.51 (0.41–0.61)
Received treatment	69	84.0	88.1	100.0 (93.8–100.0)	63.6 (30.8–89.1)	0.82 (0.67–0.97)	94.2	0.75 (0.52–0.98
**Postpartum complications**								
Postpartum hemorrhage	322	12.5	9.5	43.9 (28.5–60.3)	95.4 (92.3–97.5)	0.70 (0.62–0.77)	89.0	0.44 (0.33–0.55)
Fever	322	18.6	14.7	49.2 (36.1–62.3)	93.2 (89.5–95.9)	0.71 (0.65–0.78)	85.0	0.46 (0.35–0.57)
Retained placenta	316	5.2	4.3	52.9 (27.8–77.0)	98.7 (96.7–99.6)	0.76 (0.64–0.88)	96.2	0.58 (0.47–0.69)
Any complication	322	30.6	25.1	54.5 (44.2–64.6)	87.9 (82.9–91.9)	0.71 (0.66–0.77)	77.6	0.45 (0.34–0.56)
Received treatment	53	65.6	73.8	97.4 (86.2–99.9)	93.3 (68.1–99.8)	0.95 (0.88–1.00)	96.2	0.91 (0.64–1.18)

**Table 5:** Agreement diagnostics for select maternal pregnancy, delivery, and post-partum complications reported at 7-day and 6-month follow-up visits.

Variable	N	Reported at 7 days (%)	Reported at 6 months (%)	Sensitivity (95 CI)	Specificity (95 CI)	ROC (95 CI)	Agree-ment (%)	Kappa (95 CI)
**Pregnancy complications**								
Convulsion	321	7.5	5.0	20.8 (7.1–42.2)	96.3 (93.6–98.2)	0.59 (0.5–0.67)	90.8	0.20 (0.10–0.31)
Fever	321	13.7	6.9	22.2 (11.2–37.1)	95.4 (92.2–97.5)	0.59 (0.53–0.65)	85.2	0.22 (0.12–0.32)
Abdominal pain	320	21.9	9.3	29.6 (19.3–41.6)	96.0 (92.9–98.1)	0.63 (0.57–0.68)	81.5	0.32 (0.22–0.42)
Migraine	321	23.4	15.3	38.7 (27.6–50.6)	91.6 (87.4–94.7)	0.65 (0.59–0.71)	79.4	0.34 (0.24–0.45)
High blood pressure	306	4.8	2.9	40.0 (16.3–67.7)	99.0 (97.0–99.8)	0.69 (0.57–0.82)	96.1	0.48 (0.37–0.59)
Vaginal bleeding	321	4.4	3.7	42.9 (16.3–67.7)	97.7 (95.4–99.1)	0.69 (0.56–0.82)	95.1	0.40 (0.29–0.51)
Edema	321	18.7	15.3	66.7 (53.7–78.0)	96.6 (93.6–98.4)	0.82 (0.76–0.88)	90.8	0.68 (0.57–0.79)
Any complication	321	53.0	38.0	63.2 (55.6–70.4)	89.4 (83.4–93.8)	0.76 (0.72–0.81)	75.4	0.52 (0.41–0.62)
Received treatment	106	65.9	67.2	77.3 (66.2–86.2)	61.3 (42.2–78.2)	0.69 (0.59–0.79)	72.6	0.37 (0.18–0.56)
**Delivery complications**								
Leaking membrane 24hr	321	6.9	3.1	9.1 (1.1–29.2)	97.4 (94.9–98.9)	0.53 (0.47–0.59)	91.4	0.09 (-0.01–0.19)
Excessive bleeding	319	18.8	10.9	39.3 (27.1–52.7)	95.4 (92.1–97.6)	0.67 (0.61–0.74)	84.8	0.41 (0.31–0.52)
Prolonged labor	320	19.9	14.1	48.4 (35.8–61.3)	94.6 (91.1–97.0)	0.72 (0.65–0.78)	85.5	0.48 (0.38–0.59)
Malposition	318	3.1	3.8	54.5 (23.4–83.3)	97.7 (95.4–99.1)	0.76 (0.61–0.92)	96.3	0.48 (0.37–0.59)
Any complication	321	38.6	24.9	49.6 (40.5–58.7)	90.5 (85.5–94.2)	0.70 (0.65–0.75)	74.7	0.43 (0.32–0.53)
Received treatment	61	83.9	90.0	100.0 (93.4–100.0)	57.1 (18.4–90.1)	0.79 (0.59–0.98)	95.0	0.70 (0.46–0.94)
**Postpartum complications**								
Postpartum hemorrhage	325	12.5	7.1	27.5 (14.6–43.9)	95.8 (92.8–97.8)	0.62 (0.55–0.69)	87.4	0.28 (0.18–0.39)
Retained placenta	321	5.2	3.7	35.3 (14.2–61.7)	98.0 (95.8–99.3)	0.67 (0.55–0.78)	94.7	0.39 (0.28–0.49)
Fever	325	18.6	12.3	36.1 (24.2–49.4)	93.2 (89.4–95.9)	0.65 (0.58–0.71)	82.5	0.34 (0.23–0.44)
Any complication	321	30.6	20.5	39.2 (29.4–49.6)	87.5 (82.4–91.5)	0.63 (0.58–0.69)	72.9	0.29 (0.19–0.4)
Received treatment	37	65.3	80.0	96.0 (79.6–99.9)	33.3 (9.9–65.1)	0.65 (0.50–0.79)	75.7	0.34(0.07–0.62)

At the six-week interview, when asked to report specific complications, report of vaginal bleeding during pregnancy had the lowest indicators of agreement while edema had the highest. At the six-month interview, all complications had lower sensitivity, ROC, and kappa values than at the six-week interview, with the exception of vaginal bleeding, although none are statistically significantly different. At the six-month follow-up, convulsions had the lowest sensitivity, ROC, and kappa statistics, while edema remained the most consistently reported with the highest sensitivity, ROC and kappa statistics. Sensitivity, specificity, ROC, agreement and kappa were comparable at the six-week and six-month interview for the report of any complication during pregnancy. The statistics for the six-month interview are all marginally, though not statistically significantly, lower. The exception to this is a statistically significant reduction in the kappa statistic for reporting of fever and convulsion between the two interviews.

#### Complications during delivery

Fewer women reported complications during delivery than complications during pregnancy in all interviews. Overall, 38.8% of women reported at least one delivery complication during the seven-day interview. At the six-week visit, only 26.2% of women reported a delivery complication and this dropped to 24.9% at the six-month visit. At the six-week interview, sensitivity for all delivery complications was low, ranging from a low of 14.3% (95% CI: (0.4–57.9)) among women reporting a leaking membrane for more than 24 hours to a high of 55.4 (95% CI: 42.5–67.7) among women reporting prolonged labor. While the sensitivity of women who reported receiving treatment for a complication was 100% in both the six-week and six-month interview, the specificity was lower, with more women reporting that they received treatment in each of the follow-up visits than at the initial interview.

#### Post-partum complications

Approximately 30% of women reported experiencing at least one post-partum complication at the seven-day visit,. At the six-week interview, 25.1% of women reported a post-partum complication and by the six-month interview, this fell to 20.5%. Fever was the most commonly reported complication in all interviews. At the six-week interview, the sensitivity ranged from 43.9% (95% CI: 28.5–60.3) to 52.9% (95% CI: 27.8–77.0) for postpartum hemorrhage and retained placenta, respectively, while specificity was above 90% for all complications. The ROC curve was approximately 70% for all three post-partum complications and agreement ranged from 85.0 to 96.2. Though there were no statistically significant differences in sensitivity and specificity measurement between the six-week and six-month interviews, all of the kappa values were statistically significantly lower, and by the six-month interview, all kappas were less than .40, indicating poor agreement.

### Neonatal care and symptoms

Tables 6 and 7 below shows the numbers of neonates for whom a mother reported receiving neonatal care and any neonatal illness symptoms that occurred between birth and the first follow-up interview and the associated measures of consistency.

**Table 6:** Agreement diagnostics for select neonatal interventions and symptoms reported at 7-day and 6-week follow-up visits.

Variable	N	Reported at 7 days (%)	Reported at 6 weeks (%)	Sensitivity (95 CI)	Specificity (95 CI)	ROC (95 CI)	Agree-ment (%)	Kappa (95 CI)
**Newborn care**								
Wrapped immediate (< = 5 min)	319	55.2	49.2	67.0 (59.6–73.9)	72.7 (64.7–79.8)	0.70 (0.65–0.75)	69.6	0.39 (0.28–0.50)
Immediately placed baby on mother’s chest with skin to skin contact	319	48.9	51.1	91.7 (86.2–95.5)	87.7 (81.7–92.3)	0.90 (0.86–0.93)	89.7	0.79 (0.68–0.90)
Breastfeeding started in 1 hour	319	83.1	84.0	94.0 (90.4–96.5)	64.8 (50.6–77.3)	0.79 (0.73–0.86)	89.0	0.60 (0.49–0.71)
**Newborn illness symptoms**								
Pus in umbilicus	315	1.3	0.3	25.0 (0.6–80.6)	100.0 (98.8–100.0)	0.63 (0.38–0.87)	99.0	0.40 (0.31–0.49)
Cold	315	10.1	5.7	28.1 (13.7–46.7)	96.8 (94.0–98.5)	0.62 (0.54–0.70)	89.8	0.31 (0.20–0.41)
Fast breathing	315	1.3	0.6	33.3 (0.8–90.6)	99.7 (98.2–100.0)	0.67 (0.34–0.99)	99.0	0.40 (0.29–0.50)
Redness umbilicus	315	0.9	0.3	33.3 (0.8–90.6)	100.0 (98.8–100.0)	0.67 (0.34–0.99)	99.4	0.50 (0.40–0.59)
Eye infection	315	2.2	1.3	42.9 (9.9–81.6)	99.7 (98.2–100.0)	0.71 (0.51–0.91)	98.4	0.54 (0.43–0.64)
Vomit	315	6.6	5.7	47.6 (25.7–70.2)	97.3 (94.7–98.8)	0.72 (0.61–0.83)	94.0	0.48 (0.37–0.59)
No urine pass	315	0.6	0.3	50.0 (1.3–98.7)	100.0 (98.8–100.0)	0.75 (0.26–1.00)	99.7	0.67 (0.56–0.77)
Poor feeding	315	1.6	2.2	50.0 (6.8–93.2)	98.4 (96.3–99.5)	0.74 (0.46–1.00)	97.8	0.35 (0.25–0.46)
Difficult breathing	315	1.6	1.6	60.0 (14.7–94.7)	99.4 (97.7–99.9)	0.80 (0.56–1.00)	98.7	0.59 (0.48–0.70)
Skin lesion	315	2.5	1.6	62.5 (24.5–91.5)	100.0 (98.8–100.0)	0.81 (0.63–0.99)	99.0	0.76 (0.66–0.87)
Fever	315	1.3	2.5	75.0 (19.4–99.4)	98.4 (96.3–99.5)	0.87 (0.62–1.00)	98.1	0.49 (0.39–0.60)
Sore throat	315	1.3	1.3	75.0 (19.4–99.4)	99.7 (98.2–100.0)	0.87 (0.63–1.00)	99.4	0.75 (0.64–0.86)
Do not cry	315	0.6	0.6	100.0 (2.5–100.0)	99.7 (98.2–100.0)	1.0 (-)	99.7	0.67 (0.56–0.77)
Any illness	315	25.3	18.9	57.0 (45.3–68.1)	94.1 (90.2–96.7)	0.76 (0.70–0.81)	84.8	0.56 (0.45–0.67)

**Table 7:** Agreement diagnostics for select neonatal interventions and symptoms reported at 7-day and 6-month follow-up visits.

Variable	N	Reported at 7 days (%)	Reported at 6 months (%)	Sensitivity (95 CI)	Specificity (95 CI)	ROC (95 CI)	Agree-ment (%)	Kappa (95 CI)
**Newborn care**								
Wrapped immediate (< = 5 min)	318	55.0	43.7	57.1 (49.5–64.6)	72.7 (64.7–79.8)	0.65 (0.60–0.70)	64.2	0.29 (0.18–0.40)
Immediately placed baby on mother’s chest with skin to skin contact	318	49.1	56.9	95.5 (90.9–98.2)	82.2 (75.3–87.8)	0.89 (0.85–0.92)	88.8	0.78 (0.67–0.89)
Breastfeeding started in 1 hour	318	83.0	85.8	93.6 (89.9–96.2)	51.9 (37.8–65.7)	0.73 (0.66–0.80)	86.5	0.49 (0.38–0.60)
**Newborn complications**								
Cold	312	10.1	5.7	16.1 (5.5–33.7)	95.4 (92.2–97.5)	0.56 (0.49–0.62)	87.5	0.14 (0.04–0.25)
Vomit	312	6.6	2.5	20.0 (5.7–43.7)	98.6 (96.5–99.6)	0.59 (0.50–0.68)	93.6	0.26 (0.16–0.36)
Fast breathing	312	1.3	1.0	25.0 (0.6–80.6)	99.4 (97.7–99.9)	0.62 (0.38–0.87)	98.4	0.28 (0.17–0.39)
Skin lesion	312	2.5	2.2	37.5 (8.5–75.5)	98.7 (96.7–99.6)	0.68 (0.50–0.86)	97.1	0.39 (0.27–0.50)
Fever	312	1.3	2.5	50.0 (6.8–93.2)	98.4 (96.3–99.5)	0.74 (0.46–1.00)	97.8	0.35 (0.25–0.46)
Sore throat	312	1.3	1.9	50.0 (6.8–93.2)	98.7 (96.7–99.6)	0.74 (0.46–1.00)	98.1	0.39 (0.28–0.50)
Difficult breathing	312	1.6	1.6	60.0 (14.7–94.7)	99.3 (97.7–99.9)	0.80 (0.56–1.00)	98.7	0.59 (0.48–0.70)
Poor feeding	312	1.6	2.9	60 (14.7–94.7)	98.0 (95.8–99.3)	0.79 (0.55–1.00)	97.4	0.42 (0.31–0.52)
Hypothermia	312	0.6	1.0	100 (15.8–100)	99.7 (98.2–100)	1.0 (-)	99.7	0.80 (0.69–0.91)
Do not cry	312	0.6	1.3	100 (15.8–100)	99.4 (97.7–99.9)	1.0 (0.99–1.00)	99.4	0.66 (0.56–0.77)
Any illness	312	25.3	20.4	46.2 (34.8–57.8)	88.5 (83.7–92.3)	0.67 (0.61–0.73)	77.9	0.37 (0.26–0.48)

### Immediate neonatal care

As shown in Table 6 and Table 7, indicators of agreement remained relatively constant over the six-month period. Slightly more women at the follow-up interviews than the initial interview reported that the baby was immediately placed on the mother’s chest (48.9% at the 7-day, 51.1% at the 6-week, and 56.9% at the 6-month). While approximately equal percentages of women reported that breastfeeding started within an hour at each interview (83.2.0% at the 7-day, 84.0% at the 6-week, and 85.8% at the 6-month), the specificity was low at both the 6-week and 6-month interview (64.8% and 51.9%, respectively). Of the three immediate neonatal care indicators, reporting whether the neonate was wrapped within five minutes of delivery was the least consistent. The specificity, ROC, and agreement all showed modest declines between the 6-week and 6-month interview and the Kappa value declined from 0.39 to 0.29.

Overall, report of specific neonatal illness symptoms experienced during the first seven days was low across all interviews, with cold being the most common illness reported in all three interviews. Women reported more symptoms at the seven-day and six-week interviews than in the six month interview. Pus or redness in the umbilicus, eye infection, inability to pass urine, and difficulty breathing were both reported at the seven-day and six-week interview, but not at the six-month interview, while hypothermia was reported during the seven-day and six-month interview, but not during the six-week interview. Slightly more women reported that their infant had an illness symptom at the first interview (25.3%) than the second (18.9%) and third (20.4%). While the overall specificity was relatively high for reporting no illness, the sensitivity was low in both follow-up interviews.

### Characteristics associated with discordant reporting over time

Characteristics of the respondents were more strongly associated with inconsistent reporting of newborn health over time than maternal health (Table 8). Women age 35 and above had twice the odds of inconsistently reporting that they experienced a post-partum complication relative to women age 15–24, although the value is only marginally significant (OR: 2.03, p < .10). Primiparous women, women age 25–34 and women who received a post-natal care visit from an HEW within the first seven days had higher odds of inconsistently reporting that their infant was sick within the first seven days of life than their counterparts.

**Table 8:** Crude odds-ratio(OR) estimates from logistic regression models showing association of maternal characteristics and reporting discordant responses for complications/illness and receipt of interventions.

	At least one inconsistent report of a problem during:				At least one inconsistent report of receipt of a high impact interventions for neonate:		
	Pregnancy (OR)	Delivery (OR)	Postpartum (OR)	Neonatal (infant) (OR)	Placed naked (OR)	Wrapped (OR)	Breastfed in one hour (OR)
Facility delivery (ref: home)	0.79	1.53	1.02	.98	2.49**	1.68**	1.22
Caesarean delivery (ref: none)	0.81	1.36	0.86	0.62	2.00*	1.07	1.00
Received counseling on danger signs (ref: no)	1.27	1.31	1.33	1.15	1.53	1.39	1.17
Received PNC visit from HEW	0.61	1.35	0.97	3.15***	1.65	1.61	1.58
Parity (ref: first birth)	1.06	1.02	1.05	0.63*	0.73	0.9	0.67
Age (ref: 15–24)							
25–34	1.25	1.10	1.29	0.60*	0.61	0.78	0.62
35–39	0.83	1.48	2.03*	1.16	0.59	1.15	0.90
School (ref: none)							
Primary	1.06	0.71	0.72	1.60	2.21**	1.09	0.90
Secondary plus	0.65	1.19	0.79	1.37	2.18*	1.80**	0.57
Residence	0.98	1.47	0.91	1.05	1.46	1.39	0.70

* p < .10

** p < .05

*** p < .01

In the bivariate associations, the odds of inconsistently reporting whether the baby was placed naked on the mother’s chest were 2.5 times higher among women who delivered in a health facility compared to those who delivered at home (p < .05). Women who had a caesarean delivery had twice the odds of inconsistently reporting that the baby was placed naked on the chest compared to women who did not, although this only marginally significant (OR: 2.00, p < .10). Similarly, the odds of inconsistently reporting that the baby was wrapped was higher among facility deliveries relative to home deliveries (OR: 1.68, p < .05).

Once adjusted, most, though not all of these relationships, were no longer significant (Table 9). Age was associated with inconsistently reporting postpartum complications and women who saw an HEW for post-natal care in the first seven days had 3.29 times the odds of inconsistently reporting that their child was ill in the first seven days than women who did not see an HEW (p < .01). After adjustment, women who delivered in a facility had higher odds of inconsistently reporting whether the baby was placed naked on the chest compared to women who delivered at home (aOR 2.07, p < .10).

**Table 9:** Adjusted odds-ratio (OR) estimates from logistic regression model showing association of maternal characteristics and reporting discordant responses for complications/illness and receipt of interventions.

	At least one inconsistent report of a problem during:				At least one inconsistent report of receipt of a high impact interventions for neonate:		
	Pregnancy (OR)	Delivery (OR)	Postpartum (OR)	Neonatal (infant) (OR)	Placed naked (OR)	Wrapped (OR)	Breastfed in one hour (OR)
Facility delivery (ref: home)	0.80	1.35	1.07	0.73	2.07*	1.41	1.57
Caesarean delivery (ref: none)	0.88	1.07	0.81	0.54	1.62	0.77	1.30
Received counseling on danger signs (ref: no)	1.47	1.21	1.43	1.09	1.29	1.26	1.15
Received PNC visit from HEW	0.59	1.32	0.94	3.29***	1.29	1.43	1.47
Parity (ref: first birth)	1.01	0.97	0.78	0.64	1.24	1.21	0.66
Age (ref: 10–24)							
25–34	1.21	1.09	1.34	0.86	0.61	0.76	0.60
35–39	0.91	1.49	2.24*	1.56	0.61	1.15	0.80
School (ref: none)							
Primary	1.10	0.61	0.74	1.48	1.54	0.97	0.58
Secondary	0.61	0.81	0.66	1.40	1.21	1.42	0.37**
Residence	1.32	1.40	1.07	1.05	0.98	1.13	0.70

* p < .10

** p < .05

*** p < .01

## Discussion

This study assessed the consistency of women’s self-report of pregnancy, delivery, and postpartum complications and the consistency of maternal report of neonatal health complications over a six-month period. We note that women may have trouble identifying symptoms accurately and our estimates should not be taken as population level estimates of complications. Our intention was to assess if women *consistently* reported the experience of specific symptoms over time. Overall, we find that more women report that they experienced complications at the first interview after birth than at either the six-week or six-month interview. The specificity of the majority of complications is high, meaning that women are not likely to report a complication in later interviews that they did not report initially, however sensitivity is generally much lower and remained approximately constant over time. This indicates that while recall does decline over time, it does not seem that it declines rapidly after birth and then remains consistent once no further complications develop.

A higher percentage of women reported having received treatment in the first seven days at the follow-up interviews compared to the initial seven-day interview. Overall agreement is acceptable, but it is important to note that the samples are not identical in each interview. We could only evaluate agreement for whether treatment was sought amongst the sample of women who consistently reported that they experienced a complication in both rounds. Report of care-seeking and receipt of treatment during pregnancy and childbirth, particularly if derived from questions that rely on skip patterns regarding experience of complications, thus may not be accurate for distant events, as both showed inconsistencies even within a six-month time period. Asking whether any care was sought during delivery and in the immediate postpartum period, regardless of whether any complications were reported, may yield a more accurate estimate of care-seeking.

Few newborn illness symptoms were reported in the first seven days of life and due to small sample sizes, it limited our ability to assess the consistency of recall with precision. When aggregated to a measure of whether any symptoms at all were experienced in the first seven days of life, sensitivity was low. It may be that during the first seven days women note symptoms with higher vigilance, but if these do not later develop into serious health issues, they may be forgotten over time. Additionally, reporting on symptoms that occur within a specific time frame (e.g. within five minutes, within one hour) may become increasingly challenging to report accurately, as shown by the decline in specificity over time. This is in keeping with other studies that demonstrate low validity when reporting time bound indicators [23,31,32]. The measure of skin-to-skin contact performed the best of the three neonatal indicators, in keeping with Stanton and colleagues [23] findings that this indicator was among the most valid when self-report was compared to facility observation.

Though others studies have found that accurate reporting of complications is associated with age and parity, we found few statistically significant relationships between sociodemographic characteristics and consistent recall of maternal events [26]. While place of delivery does appear to influence whether women report complications consistently, our sample size was not large enough to confirm that these relationships were statistically significant. The literature on recall surrounding pregnancy events has relied heavily on verification using facility-based records and generally excludes women who delivered in the home. This study showed that there was a non-significant difference in recall consistency for drying and placing the baby on the chest, between women who delivered at home and women who delivered in the facility. Based on previous studies, it may be better to continue to rely on facility records for coverage of these interventions in the facility, but true population coverage rates should include these indicators in surveys for women who delivered at home. In countries with low facility delivery, such as Ethiopia, failing to include women who delivered at home may introduce a substantial degree of selection bias.

Our study is not without limitation, primarily the small sample size which resulted in few cases of several symptoms being reported, leading to large confidence intervals around several estimates. Though we attempted to address this by aggregating into whether the woman reported any or no symptoms, in doing so, we lost detail. The small sample size also limited our ability to identify any statistically significant relationships. Our study has a number of strengths, however, first among them being the longitudinal design with low loss to follow-up. Less than 5% of the initial sample was lost during the study period, and those that were did not differ substantially in background characteristics from women who were retained. Secondly, we included all women, including women who delivered at home, which reduces the selection bias associated with only sampling women who delivered in health facilities.

## Conclusions

We find that for the majority of maternal and newborn complications surrounding birth, sensitivity of self-report within six months is low, meaning women may either over-report complications immediately after birth or under-report at later points if no further complications arise. Questionnaires designed to assess care-seeking during the peripartum period should not rely on skip-patterns based on self-report of complications as these estimates may be subject to recall bias. Additionally, questions that depend on recall within time-bound periods, such as within an hour from birth or within the first seven days of life, are subject to recall bias even within short time periods. More work is needed in instrument development to improve questions that relate to very specific time periods. Much of the data around the coverage of interventions is generated from population-based surveys and our results identify an important limitation with currently fielded questions. The results of this study have important implications for the field and highlight areas where additional work in measurement is needed. Studies using longitudinal data for individual recall should be conducted in other settings to determine the replicability of our findings.

## References

[1] Lawn JE, Cousens S, Zupan J. 4 million neonatal deaths: When? Where? Why? The Lancet. 2005;365: 891–900. 10.1016/S0140-6736(05)71048-5 15752534

[2] Darmstadt GL, Bhutta ZA, Cousens S, Adam T, Walker N, de Bernis L. Evidence-based, cost-effective interventions: how many newborn babies can we save? The Lancet. 2005;365: 977–988. 10.1016/S0140-6736(05)71088-6 15767001

[3] Knippenberg R, Lawn JE, Darmstadt GL, Begkoyian G, Fogstad H, Walelign N, et al Neonatal Survival 3 Systematic scaling up of neonatal care in countries. 2005;365: 12. 10.1016/S0140-6736(05)71145-4 15781104

[4] Martines J, Paul VK, Bhutta ZA, Koblinsky M, Soucat A, Walker N, et al Neonatal survival: a call for action. The Lancet. 2005;365: 1189–1197. 10.1016/S0140-6736(05)71882-1 15794974

[5] Ronsmans C, Graham WJ. Maternal Survival 1 Maternal mortality: who, when, where, and why. 2006;368: 12. 10.1016/S0140-6736(06)69380-X 17011946

[6] Campbell OM, Graham WJ. Strategies for reducing maternal mortality: getting on with what works. The Lancet. 2006;368: 1284–1299. 10.1016/S0140-6736(06)69381-1 17027735

[7] Koblinsky M, Matthews Z, Hussein J, Mavalankar D, Mridha MK, Anwar I, et al Going to scale with professional skilled care. The Lancet. 2006;368: 1377–1386. 10.1016/S0140-6736(06)69382-3 17046470

[8] Borghi J, Ensor T, Somanathan A, Lissner C, Mills A. Mobilising financial resources for maternal health. The Lancet. 2006;368: 1457–1465. 10.1016/S0140-6736(06)69383-5 17055948

[9] Baqui AH, El-Arifeen S, Darmstadt GL, Ahmed S, Williams EK, Seraji HR, et al Effect of community-based newborn-care intervention package implemented through two service-delivery strategies in Sylhet district, Bangladesh: a cluster-randomised controlled trial. The Lancet. 2008;371: 1936–1944. 10.1016/S0140-6736(08)60835-1 18539225

[10] Kumar V, Mohanty S, Kumar A, Misra RP, Santosham M, Awasthi S, et al Effect of community-based behaviour change management on neonatal mortality in Shivgarh, Uttar Pradesh, India: a cluster-randomised controlled trial. The Lancet. 2008;372: 1151–1162. 10.1016/S0140-6736(08)61483-X 18926277

[11] Bryce J, Coitinho D, Darnton-Hill I, Pelletier D, Pinstrup-Andersen P. Maternal and child undernutrition: effective action at national level. The Lancet. 2008;371: 510–526. 10.1016/S0140-6736(07)61694-8 18206224

[12] Morris SS, Cogill B, Uauy R. Effective international action against undernutrition: why has it proven so difficult and what can be done to accelerate progress? 2008;371: 14. 10.1016/S0140-6736(07)61695-X 18206225

[13] Darmstadt GL, Kinney MV, Chopra M, Cousens S, Kak L, Paul VK, et al Who has been caring for the baby? The Lancet. 2014;384: 174–188. 10.1016/S0140-6736(14)60458-X 24853603

[14] Bhutta ZA, Das JK, Bahl R, Lawn JE, Salam RA, Paul VK, et al Can available interventions end preventable deaths in mothers, newborn babies, and stillbirths, and at what cost? The Lancet. 2014;384: 347–370. 10.1016/S0140-6736(14)60792-3 24853604

[15] Dickson KE, Simen-Kapeu A, Kinney MV, Huicho L, Vesel L, Lackritz E, et al Every Newborn: health-systems bottlenecks and strategies to accelerate scale-up in countries. The Lancet. 2014;384: 438–454. 10.1016/S0140-6736(14)60582-1 24853600

[16] Mason E, McDougall L, Lawn JE, Gupta A, Claeson M, Pillay Y, et al From evidence to action to deliver a healthy start for the next generation. The Lancet. 2014;384: 455–467. 10.1016/S0140-6736(14)60750-9 24853599

[17] Kruk ME, Kujawski S, Moyer CA, Adanu RM, Afsana K, Cohen J, et al Next generation maternal health: external shocks and health-system innovations. The Lancet. 2016;388: 2296–2306. 10.1016/S0140-6736(16)31395-2 27642020 PMC5167371

[18] Campbell OMR, Calvert C, Testa A, Strehlow M, Benova L, Keyes E, et al The scale, scope, coverage, and capability of childbirth care. The Lancet. 2016;388: 2193–2208. 10.1016/S0140-6736(16)31528-8 27642023

[19] Shaw D, Guise J-M, Shah N, Gemzell-Danielsson K, Joseph K, Levy B, et al Drivers of maternity care in high-income countries: can health systems support woman-centred care? The Lancet. 2016;388: 2282–2295. 10.1016/S0140-6736(16)31527-6 27642026

[20] Miller S, Abalos E, Chamillard M, Ciapponi A, Colaci D, Comandé D, et al Beyond too little, too late and too much, too soon: a pathway towards evidence-based, respectful maternity care worldwide. The Lancet. 2016;388: 2176–2192. 10.1016/S0140-6736(16)31472-6 27642019

[21] Bryce J, Arnold F, Blanc A, Hancioglu A, Newby H, Requejo J, et al Measuring Coverage in MNCH: New Findings, New Strategies, and Recommendations for Action. PLoS Medicine. 2013;10 10.1371/journal.pmed.1001423 23667340 PMC3646206

[22] Hancioglu A, Arnold F. Measuring Coverage in MNCH: Tracking Progress in Health for Women and Children Using DHS and MICS Household Surveys. Madise N, editor. PLoS Medicine. 2013;10: e1001391 10.1371/journal.pmed.1001391 23667333 PMC3646216

[23] Stanton CK, Rawlins B, Drake M, dos Anjos M, Cantor D, Chongo L, et al Measuring Coverage in MNCH: Testing the Validity of Women’s Self-Report of Key Maternal and Newborn Health Interventions during the Peripartum Period in Mozambique. PLoS ONE. 2013;8 10.1371/journal.pone.0060694 23667427 PMC3646219

[24] Blanc AK, Warren C, McCarthy KJ, Kimani J, Ndwiga C, RamaRao S. Assessing the validity of indicators of the quality of maternal and newborn health care in Kenya. Journal of Global Health. 2016;6 10.7189/jogh.06.010405 27231541 PMC4871064

[25] Keenan K, Hipwell A, McAloon R, Hoffmann A, Mohanty A, Magee K. Concordance between maternal recall of birth complications and data from obstetrical records. Early Human Development. 2017;105: 11–15. 10.1016/j.earlhumdev.2017.01.003 28095344 PMC5382715

[26] Stewart MK, Festin M. Validation study of women’s reporting and recall of major obstetric complications treated at the Philippine General Hospital. International Journal of Gynecology & Obstetrics. 1995;48: S53–S66. 10.1016/0020-7292(95)02320-c 7672175

[27] Seoane G, Castrillo M, O’Rourke K. A validation study of maternal self reports of obstetrical complications: Implications for health surveys. International Journal of Gynecology and Obstetrics. 1998;62: 229–236. 10.1016/S0020-7292(98)00104-0

[28] Moran AC, Kerber K, Sitrin D, Guenther T, Morrissey CS, Newby H, et al Measuring Coverage in MNCH: Indicators for Global Tracking of Newborn Care. AbouZahr C, editor. PLoS Medicine. 2013;10: e1001415 10.1371/journal.pmed.1001415 23667335 PMC3646209

[29] Central Statistical Agency (CSA), ICF. Ethiopia Demographic and Health Survey 2016. Addis Ababa, Ethiopia and Rockville, MD, USA: CSA and ICF; 2016.

[30] Zimmerman L, Olson H, PMA2020 Principal Investigators Group, Tsui A, Radloff S. PMA2020: Rapid Turn-Around Survey Data to Monitor Family Planning Service and Practice in Ten Countries: PMA2020: Rapid Turn-Around Survey Data. Studies in Family Planning. 2017;48: 293–303. 10.1111/sifp.12031 28885679 PMC6084342

[31] Blanc AK, Warren C, McCarthy KJ, Kimani J, Ndwiga C, RamaRao S. Assessing the validity of indicators of the quality of maternal and newborn health care in Kenya. Journal of Global Health. 2016;6: 1–13. 10.7189/jogh.06.010405 27231541 PMC4871064

[32] Yoder PS, Rosato M, Mahmud R, Fort A, Rahman F, Armstrong A, et al Women’s Recall of Delivery and Neonatal Care: A Study of Terms, Concepts, and Survey Questions Calverton, MD, USA: ICF Macro; 2010.

